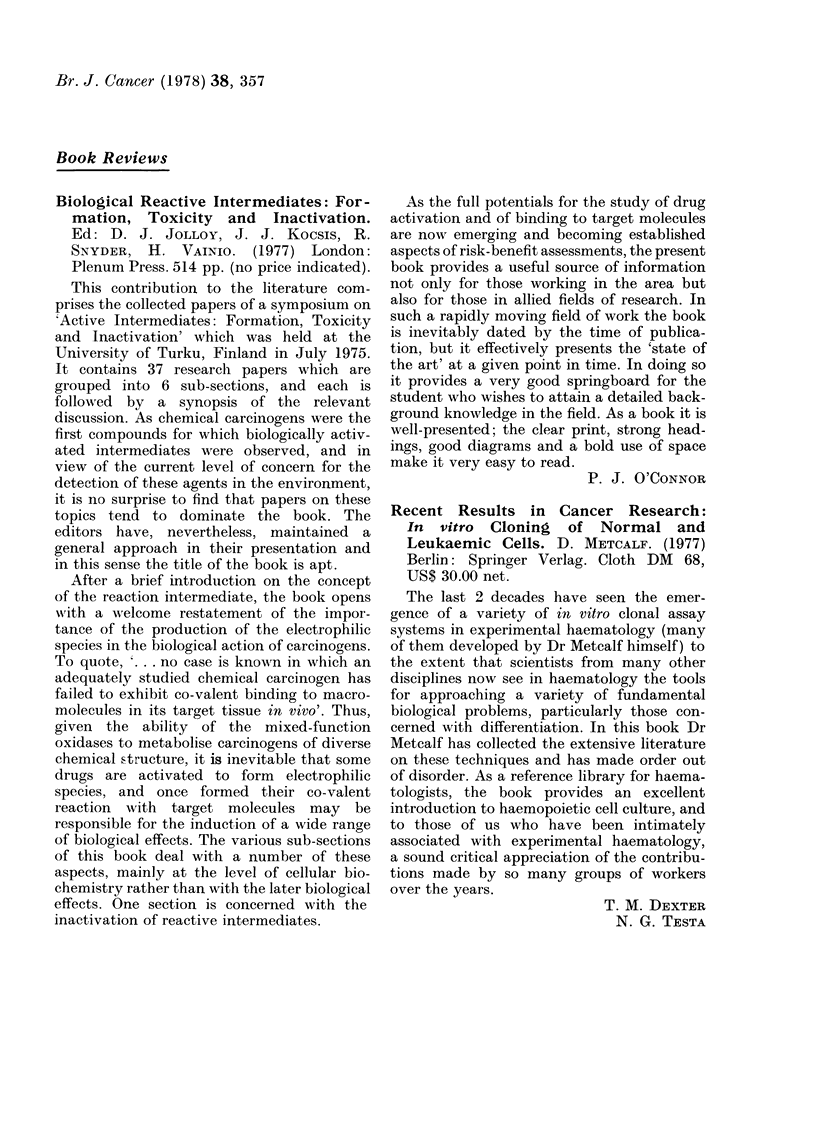# Biological Reactive Intermediates: Formation, Toxicity and Inactivation

**Published:** 1978-08

**Authors:** P. J. O'Connor


*Br. J. Cancer* (1978) **38**, 357

### Book Reviews

**Biological Reactive Intermediates: Formation, Toxicity and Inactivation.** Ed: D. J. Jolloy, J. J. Kocsis, R. Snyder, H. Vainio. (1977) London: Plenum Press. 514 pp. (no price indicated).

This contribution to the literature comprises the collected papers of a symposium on 'Active Intermediates: Formation, Toxicity and Inactivation' which was held at the University of Turku, Finland in July 1975. It contains 37 research papers which are grouped into 6 sub-sections, and each is followed by a synopsis of the relevant discussion. As chemical carcinogens were the first compounds for which biologically activated intermediates were observed, and in view of the current level of concern for the detection of these agents in the environment, it is no surprise to find that papers on these topics tend to dominate the book. The editors have, nevertheless, maintained a general approach in their presentation and in this sense the title of the book is apt.

After a brief introduction on the concept of the reaction intermediate, the book opens with a welcome restatement of the importance of the production of the electrophilic species in the biological action of carcinogens. To quote, '. . . no case is known in which an adequately studied chemical carcinogen has failed to exhibit co-valent binding to macromolecules in its target tissue *in vivo*'. Thus, given the ability of the mixed-function oxidases to metabolise carcinogens of diverse chemical structure, it is inevitable that some drugs are activated to form electrophilic species, and once formed their co-valent reaction with target molecules may be responsible for the induction of a wide range of biological effects. The various sub-sections of this book deal with a number of these aspects, mainly at the level of cellular biochemistry rather than with the later biological effects. One section is concerned with the inactivation of reactive intermediates.

As the full potentials for the study of drug activation and of binding to target molecules are now emerging and becoming established aspects of risk-benefit assessments, the present book provides a useful source of information not only for those working in the area but also for those in allied fields of research. In such a rapidly moving field of work the book is inevitably dated by the time of publication, but it effectively presents the 'state of the art' at a given point in time. In doing so it provides a very good springboard for the student who wishes to attain a detailed background knowledge in the field. As a book it is well-presented; the clear print, strong headings, good diagrams and a bold use of space make it very easy to read.

P. J. O'Connor